# Estimation of Arterial Carbon Dioxide Based on End-Tidal Gas Pressure and Oxygen Saturation

**DOI:** 10.3390/jcm7090290

**Published:** 2018-09-19

**Authors:** Raisa Rentola, Johanna Hästbacka, Erkki Heinonen, Per H. Rosenberg, Tom Häggblom, Markus B. Skrifvars

**Affiliations:** 1 Division of Intensive Care, Department of Anaesthesiology, Intensive Care and Pain Medicine, Helsinki University Hospital and University of Helsinki, 00290 Helsinki, Finland; raisa.rentola@hus.fi (R.R.); johanna.hastbacka@hus.fi (J.H.); 2 Clinical Care Solutions, Anaesthesia and Respiratory Care, 00510 Helsinki, Finland; erkki.heinonen@ge.com (E.H.); tom.haggblom@ge.com (T.H.); 3 Division of Anaesthesia, Department of Anaesthesiology, Intensive Care and Pain Medicine, Helsinki University Hospital and University of Helsinki, 00290 Helsinki, Finland; per.rosenberg@fimnet.fi; 4 Department of Emergency Care and Services, University of Helsinki and Helsinki University Hospital, 00290 Helsinki, Finland

**Keywords:** arterial carbon dioxide, mechanical ventilation, noninvasive measurement, blood gas analysis

## Abstract

Arterial blood gas (ABG) analysis is the traditional method for measuring the partial pressure of carbon dioxide. In mechanically ventilated patients a continuous noninvasive monitoring of **carbon dioxide** would obviously be attractive. In the current study, we present a novel formula for noninvasive estimation of arterial carbon dioxide. Eighty-one datasets were collected from 19 anesthetized and mechanically ventilated pigs. Eleven animals were mechanically ventilated without interventions. In the remaining eight pigs the partial pressure of carbon dioxide was manipulated. The new formula (Formula 1) is PaCO_2_ = PETCO_2_ + k(PETO_2_ − PaO_2_) where PaO_2_ was calculated from the oxygen saturation. We tested the agreements of this novel formula and compared it to a traditional method using the baseline PaCO_2_ − ETCO_2_ gap added to subsequently measured, end-tidal carbon dioxide levels (Formula 2). The mean difference between PaCO_2_ and calculated carbon dioxide (Formula 1) was 0.16 kPa (±SE 1.17). The mean difference between PaCO_2_ and carbon dioxide with Formula 2 was 0.66 kPa (±SE 0.18). With a mixed linear model excluding cases with cardiorespiratory collapse, there was a significant difference between formulae (*p* < 0.001), as well as significant interaction between formulae and time (*p* < 0.001). In this preliminary animal study, this novel formula appears to have a reasonable agreement with PaCO_2_ values measured with ABG analysis, but needs further validation in human patients.

## 1. Introduction

Mechanical ventilation is one of the most common practices in emergency and critical care settings. The primary objective is to achieve and maintain sufficient oxygen supply for organs and an adequate clearance of carbon dioxide (CO_2_) from the body [1]. Considering CO_2_, both hypocapnia and hypercapnia should be avoided [2]. Frequent control of the arterial partial pressure of carbon dioxide (PaCO_2_) is needed especially for patients with brain injury because carbon dioxide dilates the cerebral blood vessels and may, therefore, increase intracranial pressure [3]. Hypocapnia, on the other hand, causes cerebral vasoconstriction and may lead to regional cerebral ischemia, and has been shown to worsen outcomes in patients with traumatic brain injury [4,5]. Nonetheless, unintentional hypocapnia commonly occurs in clinical practice [6].

Arterial blood gas (ABG) analysis is the reference method for monitoring adequate ventilation in mechanically ventilated patients [4]. Consequently, ABG analysis is the most frequent laboratory test in the intensive care unit (ICU) [7]. The average number of ABG samples varies; in a study from our department, eight samples were taken during the first 24 h in patients treated following cardiac arrest [8]. ABG analysis is also difficult or impossible in some situations, such as in the prehospital setting and during patient transportation [9].

A noninvasive method for estimating arterial CO_2_ would have obvious implications for clinical practice. A commonly used strategy, under stable conditions, is to calculate the gap between end-tidal CO_2_ (ETCO_2_) and PaCO_2_ under the assumption that the gap remains constant, and use the continuous ETCO_2_ value for arterial CO_2_ estimation over time. However, because the gap may change, this method is likely to become inaccurate with time. In this experimental animal study, we tested a novel formula for calculating arterial carbon dioxide partial pressure. The formula utilizes noninvasive patient gas parameters readily available in ventilated patients and could provide an alternative way to determine PaCO_2_ value without ABG analysis. We hypothesized that this formula would outperform traditional end-tidal CO_2_ (ETCO_2_)-based PaCO_2_ prediction, also in situations involving concomitant changes in ventilation and perfusion. 

## 2. Materials and Methods

The current study consists of an analysis of data collected during a series of animal experiments undertaken at the Research and Development Unit of the Helsinki University Hospital, Helsinki, Finland between September 2015 and September 2016. The animal experiments were a part of a developmental project where a novel adaptive ventilation control system was applied on traditional pressure- (PCV) and volume-controlled (VCV) mechanical ventilation and on pressure support (CPAP-PSV) of spontaneously triggered breathing. The developmental project was conducted for internal purposes only, and it will not be published in a scientific journal. The study protocol was approved by the National Animal Experiment Board (ESAVI/1801/04.10.07/2015; Hämeenlinna, Finland) and by the Hospital Board of Helsinki University Hospital (17 June 2015).

### 2.1. Study Subjects

Nineteen Landrace pigs of both genders were used for the experiments. The average weight of the pigs was 28.7 ± 10.1 kg (range 15.5–47.0 kg). All animals were fasted overnight but had free access to water. Twenty minutes prior to testing, the animals were premedicated with intramuscular medetomidine (80 μg/kg) and ketamine (10 mg/kg). An ear vein was cannulated, and anesthesia was induced using bolus doses of propofol (4–5 mg/kg). After tracheal intubation, the pigs were mechanically ventilated using either volume control ventilation (VCV), pressure control ventilation (PCV), or continuous positive airway pressure combined with pressure support ventilation (CPAP + PSV) mode. A five-lead electrocardiogram and peripheral capillary oxygen saturation through a pulse oximeter were recorded continuously. Respiratory gases (fraction inspired carbon dioxide [FiCO_2_], ETCO_2_, fraction inspired oxygen [FIO_2_] and end tidal oxygen [ETO_2_]) were measured with an S/5 Anaesthesia Monitor (GE Healthcare, Helsinki, Finland) [10]. Blood gases were measured using an epoc^®^ Blood Analysis System (Alere, Waltham, MA, USA).

Anesthesia was maintained with sevoflurane, and analgesia was provided by boluses of fentanyl. Depth of anesthesia was monitored using EntropyTM in addition to visual observation. The femoral artery was cannulated for blood pressure monitoring and arterial blood sampling, and blood pressure was measured invasively. A central venous catheter was inserted in some cases for monitoring of central venous pressure, infusion of intravenous fluids and medication, and for creating an air embolism as described below. After completing the test, the animals were euthanized with a bolus of potassium chloride. Baseline characteristics, physiological parameters, and data on mechanical ventilation are shown in Supplementary Tables S1 and S2.

### 2.2. Experimental Procedures

The first set of ABG measurements was taken after induction of anesthesia under stable hemodynamical conditions in normoventilated animals. In two animals, the testing included different depths of anesthesia and awakening with spontaneous breathing. In one animal, the procedures included mildly elevated abdominal pressure and awakening. In 10 animals (pigs 9–19), the only intervention undertaken was elevation of intra-abdominal pressure by inflating carbon dioxide into the abdominal cavity to a pressure of 15 mmHg. In eight animals (pigs 1–8), various procedures were undertaken in an attempt to manipulate the arterial content of carbon dioxide.

These included:

- Inducing metabolic acidosis with an infusion of lactate (30–32 mmol) followed by infusion of 1 mL/kg sodium bicarbonate to increase the CO_2_ content;
- Experimental air embolism with the injection of 60 mL of air into the central vein;
- Lung injury with the injection of 0.9% sodium chloride endobronchially;
- Endobronchial ventilation with blocking of the left bronchus with the inflated balloon of a Swan–Ganz catheter balloon acting as a bronchial blocker;
- Trendelenburg position.

Considering further ABG samples, a 15 min stabilization period was allowed after each intervention in order to reach hemodynamic stability before sampling. The only exception was in a case where an air embolus was injected: in a situation of rapidly deteriorating hemodynamics, stabilization was not possible and the sample was taken without a stabilization period. The outline of the experimental protocol is shown in the Supplemental Figure S1a.

### 2.3. Estimation of Arterial CO_2_ Partial Pressure

Synchronously with the measurement of ABG we obtained data on respiratory gases, i.e., FICO_2_, ETCO_2_, FIO_2_, and ETO_2_, and used these in a formula for the estimation of PaCO_2_.

The novel formula (Formula 1) used for prediction of PaCO_2_ is defined as

PaCO_2_ = PETCO_2_ + k(PETO_2_ − PaO_2_).

The relationship factor k is defined by fitting unpublished datapoints for the minimal difference between the blood gas measured PaCO_2_ and the Formula 1 calculated value. PETCO_2_ and PETO_2_ are the respective measured dry gas end-tidal volume % values converted to body temperature and pressure saturated partial pressures. For this purpose, we utilized one datapoint from each of 190 emergency patients breathing room air and of 43 ICU ventilated patients recently treated in our university hospital. This fitting gave the value k = 0.035 for samples when ETO_2_ ≤ 60 kPa and k = 0.01 when ETO_2_ > 60 kPa. PaCO_2_ and PaO_2_ are the arterial CO_2_ and O_2_ partial pressures, respectively, and PETCO_2_ and PETO_2_ are the end-tidal CO_2_ and O_2_ pressures. The estimation of PaO_2_ was based on values from the oxygen dissociation curve assuming a normal pH level and is presented in Supplementary Materials Table S3 [11]. Despite differences in the oxygen dissociation curve between humans and pigs, we did not use a porcine-specific method for measuring hemoglobin saturation, possibly overestimating the PaO_2_ level [12].

In addition to Formula 1, we used another method to estimate PaCO_2_. First, the difference between measured PaCO_2_ and ETCO_2_ was calculated from the first available ABG and the corresponding capnography value. This difference was added to the subsequent ETCO_2_ values at the times when subsequent ABG samples were taken (Formula 2: PaCO_2_ = ETCO_2_ + (PaCO_2_ − ETCO_2_)). We compared this method to our novel formula (Formula 1).

Formulae:

Formula 1 PaCO_2_ = PETCO_2_ + k(PETO_2_ − PaO_2_)

Formula 2 PaCO_2_ = ETCO_2_ + (PaCO_2_ − ETCO_2_).

### 2.4. Statistical Analyses

Categorical variables are expressed as a number and percentage. Normally and non-normally distributed continuous variables are expressed as mean (±SD) and median (range), respectively. To demonstrate the agreement between the estimated and measured values of blood PaCO_2_, we created Bland–Altman plots [13] with 95% confidence intervals. Mean differences between the measured PaCO_2_ and both formulae with the limits of agreement and their respective 95% confidence intervals were calculated. The standard deviations of the differences with their standard errors (SE) were calculated. Within-subject and between-subject variances (WSW and BSV) and intra-class correlations (τ) were calculated for both formulae as well as the repeatability coefficients. The data were not normally distributed (Kolmogorov–Smirnov test, *p* value < 0.001). The normality of the distribution of the differences between the measured and estimated values were tested using the Kolmogorov–Smirnov test (*p* values 0.218 and 0.138). The two formulae were compared, regarding the difference between the estimated and actual PaCO_2_ value over time, with a mixed linear model, with subjects treated as a random effect and method, time, and their interaction as fixed effects. For the Bland–Altman analysis including the bias with ±SE and the limits of agreement with 95% confidence intervals we used the freely available software created by Olofsen et al. [14].

Other analyses were performed using Statistical Package for Social Sciences version 24 software (SPSS Inc., Chicago, IL, USA), SAS version 9.4 (SAS Institute Inc., Cary, NC, USA), and SPSS for Windows (Version 22.0, IBM, Armonk, NY, USA).

## 3. Results

In total, 81 datasets were collected from 19 pigs. Baseline characteristics and data on mechanical ventilation are shown in the Supplementary Tables S2 and S3.

### 3.1. Difference between Estimated and Measured Values

**Figure 1:**
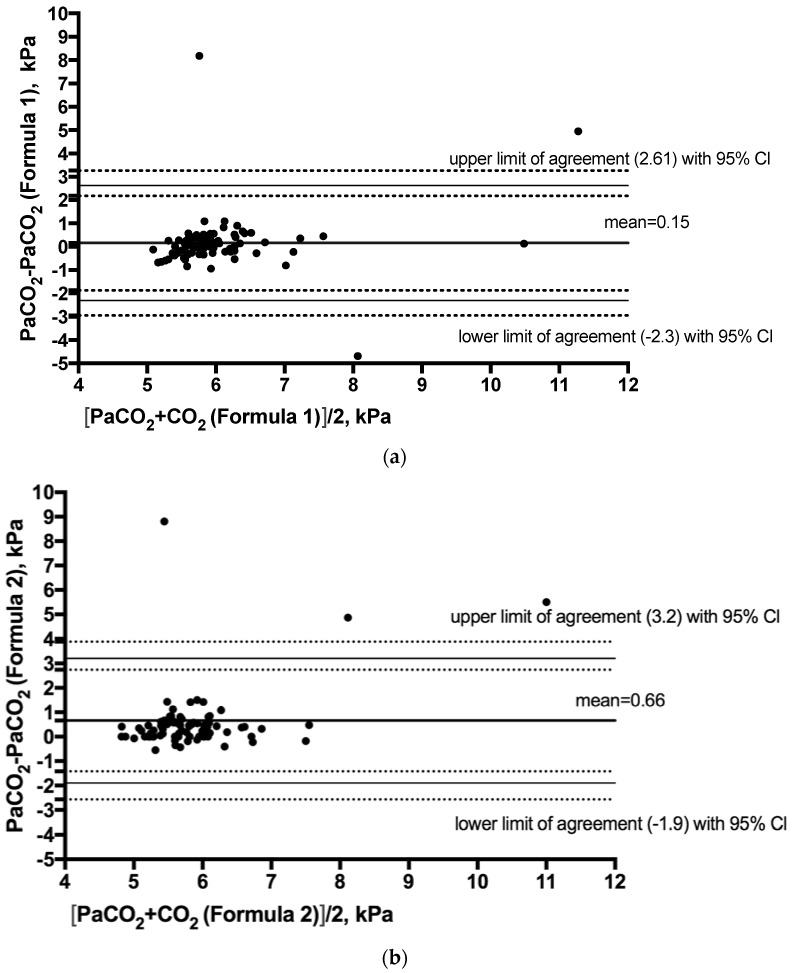
Bland–Altman plots with 95% limits of agreement with 95% confidence intervals (CIs) demonstrating (**a**) the difference between the arterial partial pressure of carbon dioxide (PaCO_2_) (Formula 1) and measured PaCO_2_, and (**b**) the difference between PaCO_2_ (Formula 2) and measured PaCO_2_.

In our study, we had three clear outliers. In two cases, mean arterial pressures (MAPs) were below 60 mmHg, and in one case, the MAP was decreasing rapidly and reached 25 mmHg two minutes after arterial blood sampling. The mean difference between measured and calculated (Formula 1) PaCO_2_ was 0.15 kPa (SE ± 0.17). The SD of the differences was 1.26 (SE ± 0.1). Formula 1 overestimated PaCO_2_ in 42% of cases and underestimated PaCO_2_ in the remaining 58%. Excluding extreme outliers, the mean difference (Formula 1) was 0.055 kPa (SD 0.44; SE ± 0.05). The mean difference between measured and calculated (Formula 1) PaCO_2_ before interventions was −0.06 kPa, (SD 0.49, SE ± 0.09). The mean difference for Formula 2 was 0.66 kPa (SE ± 0.18) and the SD of these differences was 1.3 kPa (SE ± 0.1). Excluding extreme outliers, the mean difference (Formula 2) was 0.42 kPa, and the SD of these differences was 0.44 kPa (SE ± 0.57). Formula 2 underestimated PaCO_2_ in 87% of cases and overestimated PaCO_2_ in the remaining 13%. The Bland–Altman plots with limits of agreement and their 95% confidence intervals demonstrating the agreement between PaCO_2_ (Formula 1) and measured PaCO_2_ and between PaCO**_2_** (Formula 2) and measured PaCO_2_ are presented in Figure 1a,b.

**Figure 2:**
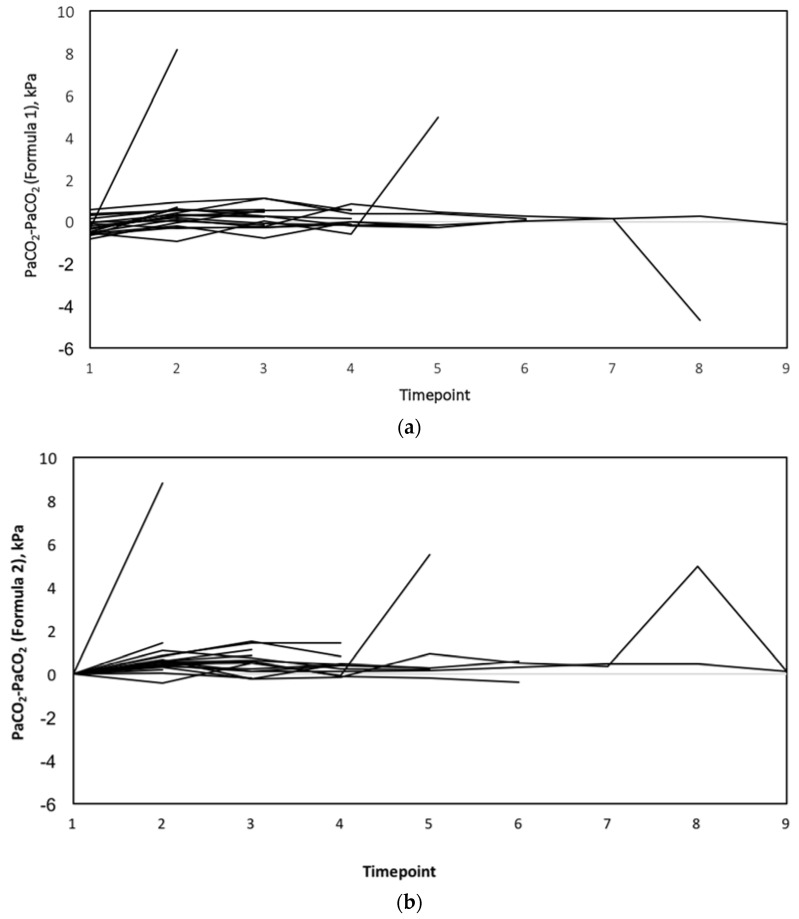
(**a**) The differences between estimated PaCO_2_ (Formula 1) and measured CO_2_ values at each time point. (**b**) The differences between estimated PaCO_2_ (Formula 2) and measured CO_2_ values at each time point.

With linear mixed-model analysis including all values, there were no statistically significant differences between the two methods for estimating PaCO_2_ (*p =* 0.057), but there was significant interaction between methods and time (*p =* 0.014). In a model excluding outliers, the difference between the methods was statistically significant (*p* < 0.001), and there was significant interaction between methods and time (*p* < 0.001). The differences between PaCO_2_ and PaCO_2_ estimated using Formulae 1 and 2 over time points are presented in Figure 2a,b.

### 3.2. Within-Subject and Between-Subject Variance and Intra-Class Correlation

**Figure 3:**
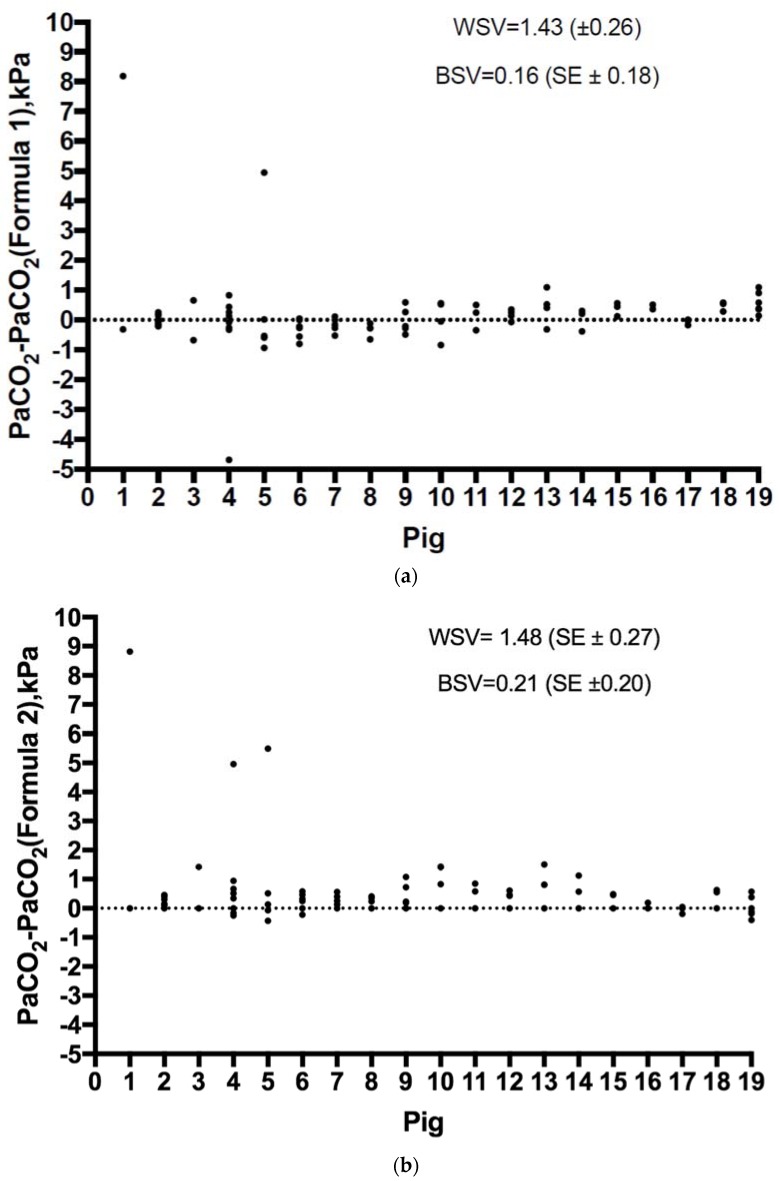
(**a**) The difference between PaCO_2_ (Formula 1) and measured PaCO_2_ for each animal. (**b**) The difference between PaCO_2_ (Formula 2) and measured PaCO_2_ for each animal*.* WSV, within-subject variance; BSV, between-subject variance.

The within-subject variances (WSV) for Formula 1 and Formula 2 were 1.43 (SE ± 0.26) and 1.48 (SE ± 0.27), respectively, and the between-subject variances were 0.16 (SE ± 0.18) and 0.21 (SE ± 0.20), respectively. The intra-class correlations (τ = ratio of BSV and total variance) for Formula 1 and Formula 2 were 0.1 (SE ± 0.1, Spearman’s ρ 0.31, SE ± 0.12) and 0.13 (SE ± 0.1, Spearman’s ρ 0.05, SE ± 0.15), respectively. The differences for Formula 1 and Formula 2 in terms of individual measurements regarding each animal and according to the interventions are presented in Figure 3a,b.

### 3.3. Performance in Animals with Metabolic Acidosis

The difference between PaCO_2_ and estimated PaCO_2_ by Formula 1 was largest in two pigs when metabolic acidosis was simulated (mean differences 4.25 and 1.4 kPa). The smallest difference was found in the pig ventilated in the Trendelenburg position (mean difference 0.21 kPa).

### 3.4. Performance in Animals under Stable Conditions and in Comparison with the End-Tidal CO_2_

The mean difference between PaCO_2_ and estimated PaCO_2_ by Formula 1 in animals with CO_2_ insufflation as the only intervention (9–19) was 0.24 kPa (SD 0.5; SE ± 0.08). The corresponding mean difference for Formula 2 in these animals was 0.4 kPa (SD 0.54; SE ± 0.09). The Bland–Altman plots with 95% confidence intervals demonstrating the agreement between PCO_2_ (Formula 1) and measured PaCO_2_ and between PaCO_2_ (Formula 2) and measured PaCO_2_ in animals 9–19 are presented in Supplementary Material Figure S2a,b.

The mean difference between measured PaCO_2_ and end-tidal CO_2_ was 1.1 kPa (SD 1.3; SE ± 0.14). The difference between end-tidal CO_2_ and PaCO_2_ over time is shown in the Supplementary Materials in Figure S3, and the Bland–Altman plot with 95% confidence intervals demonstrating the agreement between PaCO_2_ and ETCO_2_ is presented in the Supplementary Materials in Figure S4.

In an additional analysis of 26 measurements obtained prior to any intervention aiming at modifying carbon dioxide, the mean difference between the estimated PaCO_2_ with Formula 1 was 0.25 kPa (SD 0.4; SE ± 0.08), The corresponding difference for ETCO_2_ and PaCO_2_ was 0.83 kPa (SD 0.5; SE ± 0.1). The corresponding Bland–Altman plots with 95% confidence intervals are presented in the Supplementary Material Figure S5a,b. 

## 4. Discussion

In this study, we tested the agreement of two noninvasive methods to estimate PaCO_2_. We found that our novel formula, which uses information from respiratory gas measurements available during mechanical ventilation, appears more accurate in a hemodynamically stable state than the commonly used ETCO_2_ gap method. After controlled validation in clinical studies, this novel formula may have clinical implications in cases where PaCO_2_ control is important but where ABG analysis is difficult, such as during patient transport. To the best of our knowledge, no studies on the estimation of PaCO_2_ using readily available respiratory gas values and oxygen saturation have been published.

In previous studies the acceptable range of agreement has been set at 1 kPa between different methods of estimating PaCO_2_ [15]. Importantly, the clinically significant difference may vary depending on the situation; e.g., for neurosurgical patients with critically elevated intracerebral pressure, changes of more than 0.5 kPa in PaCO_2_ values may be extremely significant. One key point of the novel formula is the definition of the factor k. It has been developed from data in patients who were breathing room air, so it is likely that additional data from subjects receiving supplemental oxygen may refine this value further, especially in relationship to increased oxygen pressures.

Previous studies regarding a correlation between end-tidal carbon dioxide (ETCO_2_) and PaCO_2_ have shown conflicting results. On one hand, Razi and colleagues found a strong correlation between ETCO_2_ and PaCO_2_ in 87 critically ill mechanically ventilated patients [16]. On the other hand, Kerr and colleagues, in a study on head trauma patients, concluded that the clinical validity of estimation of PaCO_2_ with ETCO_2_ was not sufficient in patients with respiratory distress or during spontaneous breathing [17]. According to our results, when using a method assuming a stable gap between ETCO_2_ and PaCO_2_, the value of PaCO_2_ is underestimated, which might lead to insufficient ventilation. Probably, the gradient between ETCO_2_ and PaCO_2_ does not remain constant when a patient’s clinical condition changes; this decreases the accuracy. Belenkiy and colleagues used volumetric capnography (Vcap) in a porcine model of chest trauma and showed that the difference between the estimated and measured PaCO_2_ values changed only slightly [18].

Transcutaneous CO_2_ (tc-CO_2_) monitoring has been used especially in neonates and infants [19]. The accuracy of tc-CO_2_ has varied in studies and, for example, during low flow shock, tc-CO_2_ is not a consistently accurate reflection of PaCO_2_ [20] and is dependent on microvascular circulation [21]. Estimations based on end-tidal carbon dioxide may be sufficient in selected patients, assuming the clinical condition is stable, which is not the case in many critically ill patients in the ICU or prehospital setting.

In this study, the interventions undertaken to influence CO_2_ in a few cases caused cardiovascular and respiratory collapse. As expected and due to the rapid decrease in cardiac output (CO) and lung perfusion, this resulted in much poorer accuracy of both formulae estimating PaCO_2_. Ornato et al. showed that the relationship between CO and ETCO_2_ was more logarithmic than linear, resulting in big changes in ETCO_2_ when changes in CO were rather small [22]. During low or high CO, ETCO_2_ seems to be a poor indicator of PaCO_2_. In prehospital settings, ETCO_2_ cannot estimate PaCO_2_, since the gradient between PaCO_2_ and ETCO_2_ varies greatly. The difference is especially notable in patients with hypocapnia [23]. Similar results have been shown with other methods using ETCO_2_ for the estimation of PaCO_2_. Mechanically ventilated patients with severe trauma or burns have poorer correlation between ETCO_2_ and PaCO_2_ in prehospital settings, with acidosis and poor outcome as consequences [24]. When FIO_2_ approaches 100%, the reliability of the novel formula is impaired. In one case during our study, a tube occlusion problem occurred, and the difference between PaCO_2_ and calculated PaCO_2_ was near 5 kPa. In addition to the obvious unreliability of ETCO_2_ associated with tube occlusion, the high difference was partly due to high FIO_2_, as pulse oximetry is a poor indicator of PaO_2_ [25]. Indeed, our results are preliminary and cannot be generalized at this stage to other populations. In an ongoing further study, our aim is to test the accuracy of the presented formula with a large group of mechanically ventilated ICU patients with and without respiratory failure.

If this novel formula is validated in clinical studies, a built-in algorithm in patient monitoring could offer continuous information about a patient’s estimated PaCO_2_ level. This could be valuable in settings without the possibility of ABG analysis. Indeed, our results show that the novel algorithm was more accurate than estimating carbon dioxide with end-tidal CO_2_, which is commonly used in the prehospital setting. Eventually, ventilators that keep patients’ CO_2_ levels stable by modifying tidal volume and pressure may be developed; using an equation embedded in the ventilator for estimating arterial CO_2_ tension is one possible practical application.

Our study has some important limitations. First, the number of individual animals subjected to different experiments was small and, thus, the respiratory and hemodynamic conditions were not standardized. Second, ABG analyses were not taken at specified time points, and the number of ABG analyses varied. Third, as mentioned previously, remarkable changes in cardiac output result in a poor capacity to estimate PaCO_2_ with the traditional method. In this study we did not measure cardiac output; hence, it is unclear to what extent it applies to the new algorithm. Fourth, we did not use hyperventilation or very high FIO_2_ in the experiments. At this point, it is uncertain how the new algorithm functions for patients with hypocapnia or in situations where a high FIO_2_ is used. In the setup of the original study, the goal was normoventilation while manipulating PaCO_2_ in various interventions. In addition, the determination of factor k used in the algorithm may change with more datapoints. In situations where ETO_2_ is remarkably high, for example, massive shunting in lungs, the algorithm does not function. Finally, despite differences in the oxygen dissociation curve between humans and pigs, we did not use a porcine-specific method for measuring hemoglobin saturation, possibly overestimating the PaO_2_ level [25]. Finally, the samples were obtained in animals ventilated with a novel adaptive ventilation control system. However, this novel ventilation system uses common ventilation modes that are not likely to influence the end-tidal concentrations of either oxygen or carbon dioxide any differently than other currently used mechanical ventilators. Nonetheless, the inherent limitations of the study design are not likely to overestimate the accuracy of the novel algorithm, but the opposite. Some of the limitations, such as lack of standardization of the experiments, render the study sample more similar to a clinical real-life scenario than a tightly controlled experimental setting would have done. 

## 5. Conclusions

We present a new formula for estimating PaCO_2_ noninvasively using pulse oximetry and measurement of inspired and expired gas fractions of CO_2_ and O_2_. In this preliminary animal study, this formula appears to have a reasonable agreement with PaCO_2_ values measured with ABG analysis, but it needs further validation in human patients.

## Supplementary Materials

The following are available online at http://www.mdpi.com/2077-0383/7/9/290/s1, Figure S1: The flowchart of experimental protocol, Figure S2a: The Bland-Altman plot demonstrating the difference between estimated PaCO_2_ (Formula 1) and measured PaCO_2_ with 95% confidence intervals in animals 9–19, Figure S2b: The Bland-Altman plot demonstrating the difference between estimated PaCO_2_ (Formula 2) and measured PaCO_2_ with 95% confidence intervals in animals 9–19, Figure S3: The differences between measured PaCO_2_ and end-tidal CO_2_ values at each time point, Figure S4: The Bland-Altman plot demonstrating the difference between measured PaCO_2_ and end-tidal carbon dioxide (ETCO_2_) with 95% confidence intervals, Figure S5a: The Bland-Altman plot demonstrating the difference between estimated PaCO_2_ (Formula 1) and paCO_2_ with 95% confidence intervals in 26 cases prior to any intervention aiming at modifying CO_2_, Figure S5b: The Bland-Altman plot demonstrating the difference between end-tidal carbon dioxide (ETCO_2_) and paCO_2_ with 95% confidence intervals in 26 cases prior to any intervention aiming at modifying CO_2_, Table S1: Weight, interventions performed, number of ABG analyses, values of measured PaCO_2_ and pH values in the study subjects. The numbering of animals are based on timing, with the first experimental animal labelled number one and the last number nineteen, Table S2: Hemodynamic variables, etCO_2_, etO_2_, PaO_2_ and PaO_2_/FiO_2_ ratios of each animal during the experiment, Table S3: Estimation of PaO_2_ based on values of oxygen-dissociation curve.
