# Supplementary material for: Estimation of Arterial Carbon Dioxide Based on End-Tidal Gas Pressure and Oxygen Saturation
Source: J Clin Med. 2018 Sep 19;7(9):290. doi: 10.3390/jcm7090290 (PMC6162395; doi:10.3390/jcm7090290)
Supplement: Supplementary file 1 [file jcm-07-00290-s001.pdf]

SUPPLEMENTARY MATERIAL

Estimation of arterial carbon dioxide based on end-tidal gas pressure and oxygen saturation

Raisa Rentola [1], Johanna Hästbacka [1], Erkki Heinonen [2], Per H Rosenberg [3], Tom Häggblom [2], and Markus B Skrifvars [1,*]

[1] Division of Intensive Care, Department of Anaesthesiology, Intensive Care and Pain Medicine, Helsinki University Hospital and University of Helsinki, Helsinki 00290, Finland; raisa.rentola@hus.fi; johanna.hastbacka@hus.fi
[2] Clinical Care Solutions, Anaesthesia and Respiratory Care, Helsinki 00510, GE Healthcare Finland OY; erkki.heinonen@ge.com; tom.haggblom@ge.com
[3] Division of Anaesthesia, Department of Anaesthesiology, Intensive Care and Pain Medicine, Helsinki University Hospital and University of Helsinki, Helsinki 00290 Finland; per.rosenberg@helsinki.fi
* Correspondence: markus.skrifvars@hus.fi; Tel.: +358504272424

Figure S1. The flowchart of experimental protocol.

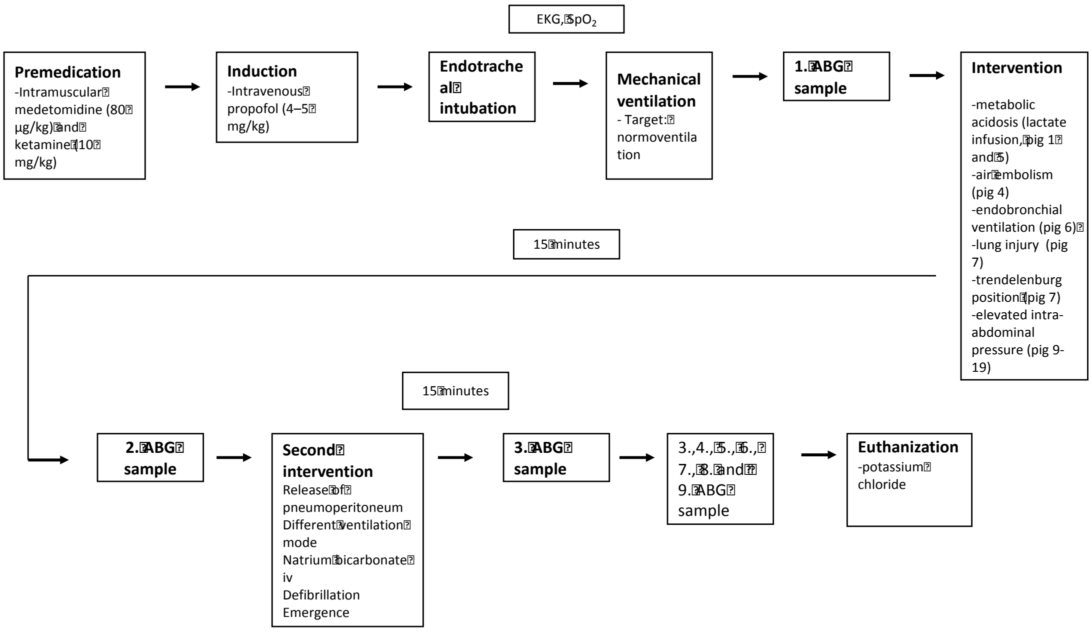

Figure S2a. The Bland-Altman plot demonstrating the difference between estimated PaCO2 (Formula 1) and measured PaCO2 with 95% confidence intervals in animals 9-19.

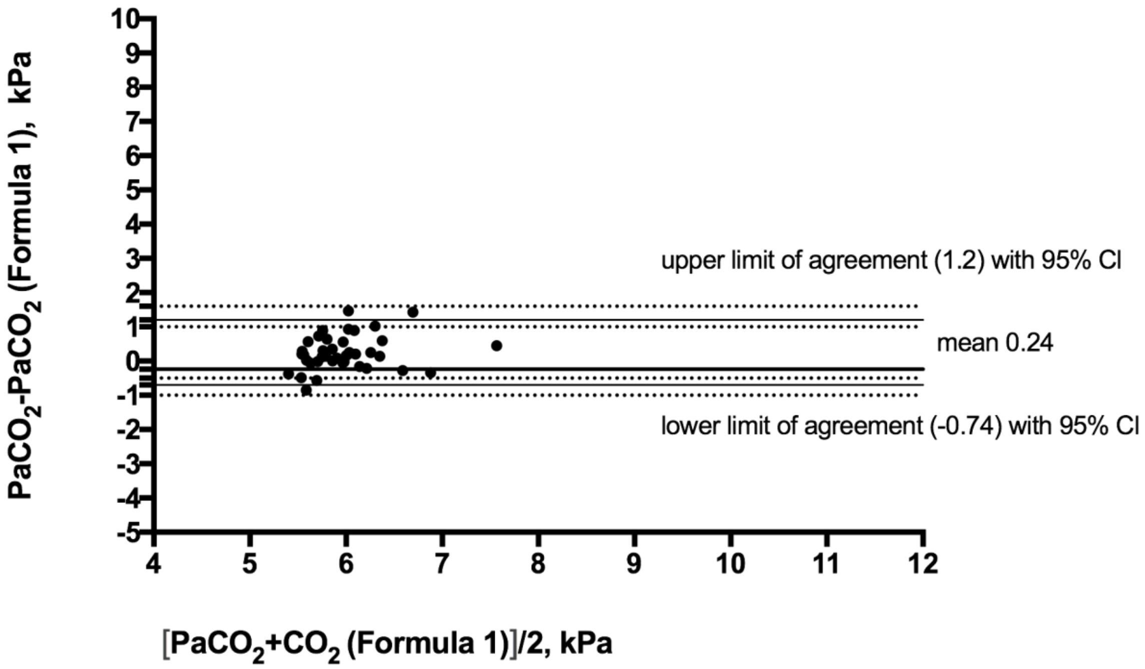

Figure S2b. The Bland-Altman plot demonstrating the difference between estimated PaCO2 (Formula 2) and measured PaCO2 with 95% confidence intervals in animals 9-19.

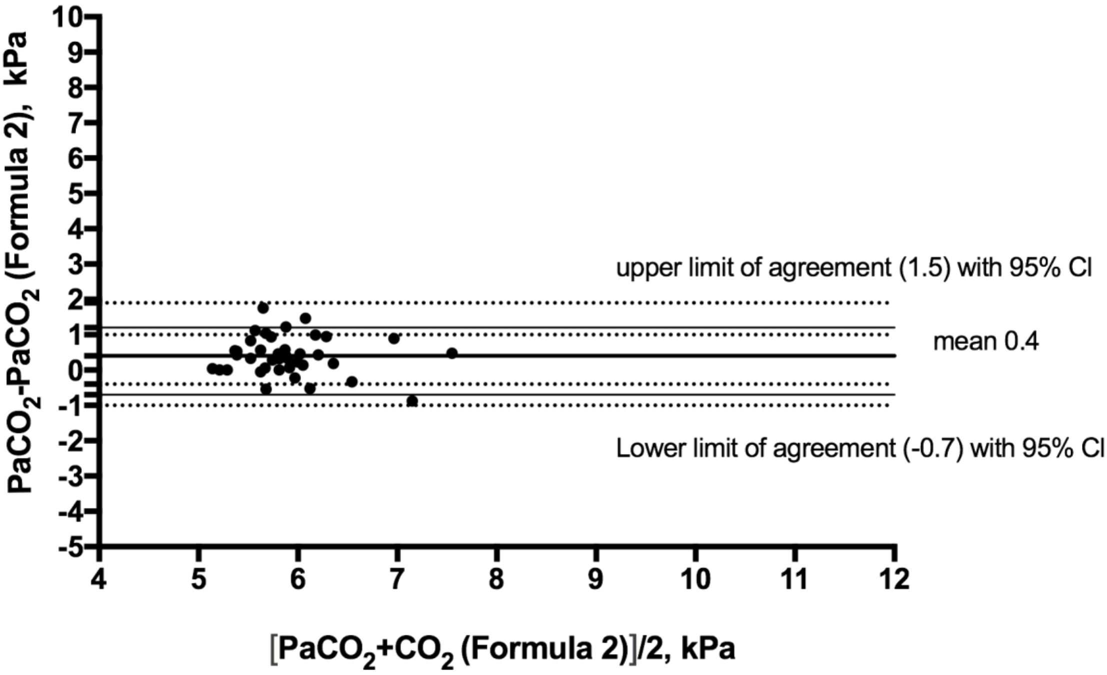

Figure S3. The differences between measured PaCO2 and end-tidal CO2 values at each time point.

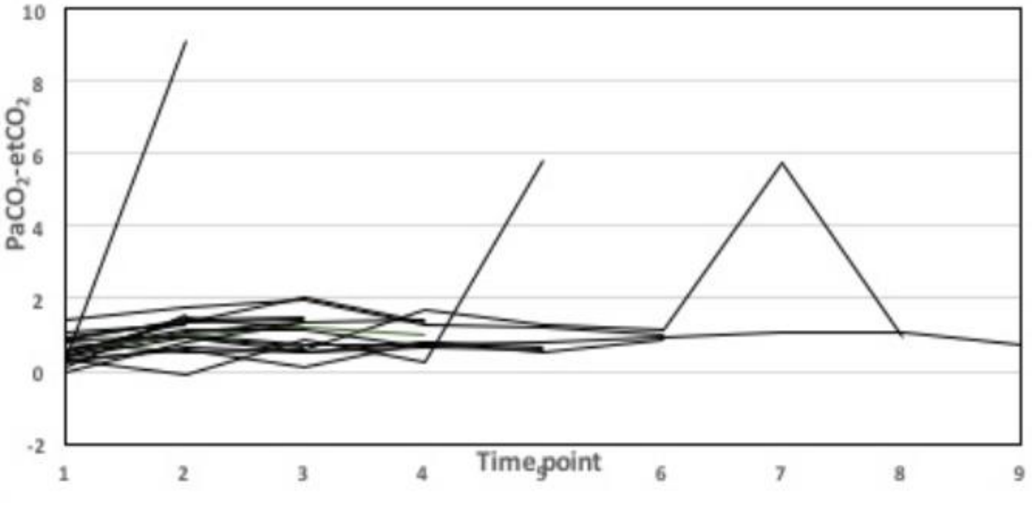

Figure S4. The Bland-Altman plot demonstrating the difference between measured $PaCO_2$ and end-tidal carbon dioxide ($ETCO_2$) with 95% confidence intervals.

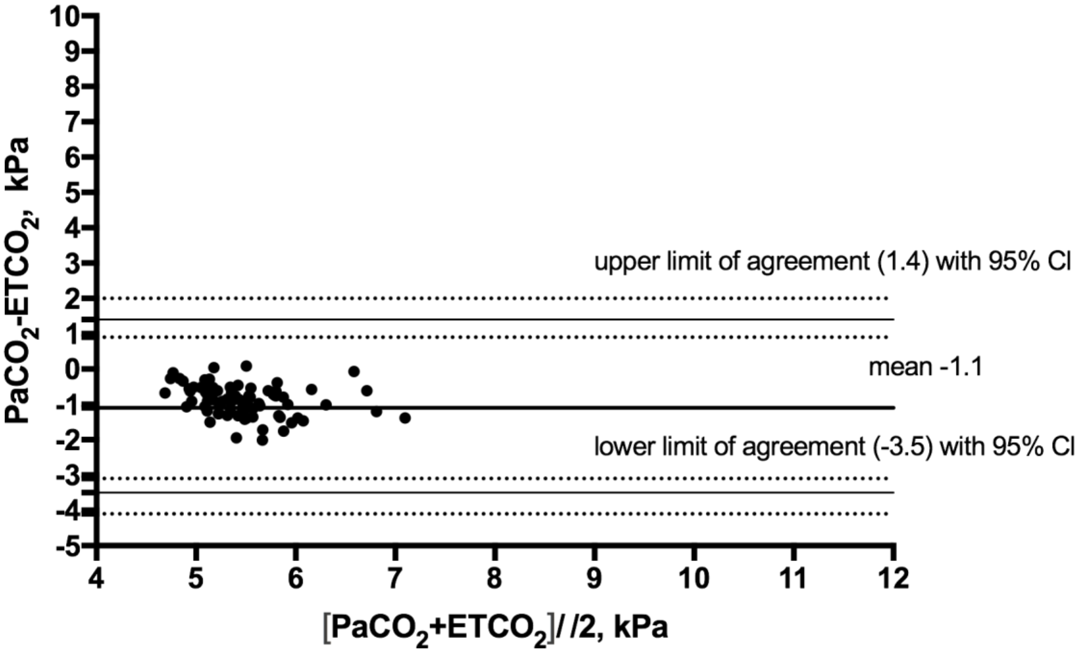

Figure S5a. The Bland-Altman plot demonstrating the difference between estimated $PaCO_2$ (Formula 1) and $paCO_2$ with 95% confidence intervals in 26 cases prior to any intervention aiming at modifying CO_2.

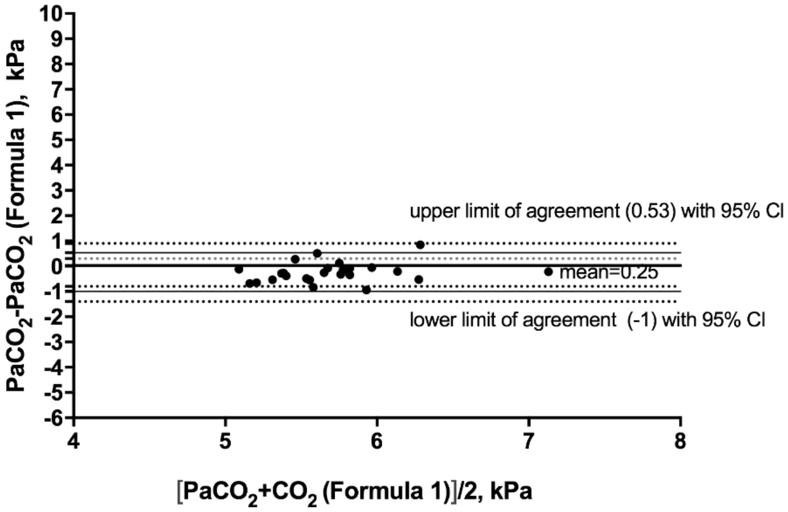

Figure S5b. The Bland-Altman plot demonstrating the difference between end-tidal carbon dioxide ($ETCO_2$) and $paCO_2$ with 95% confidence intervals in 26 cases prior to any intervention aiming at modifying CO2.

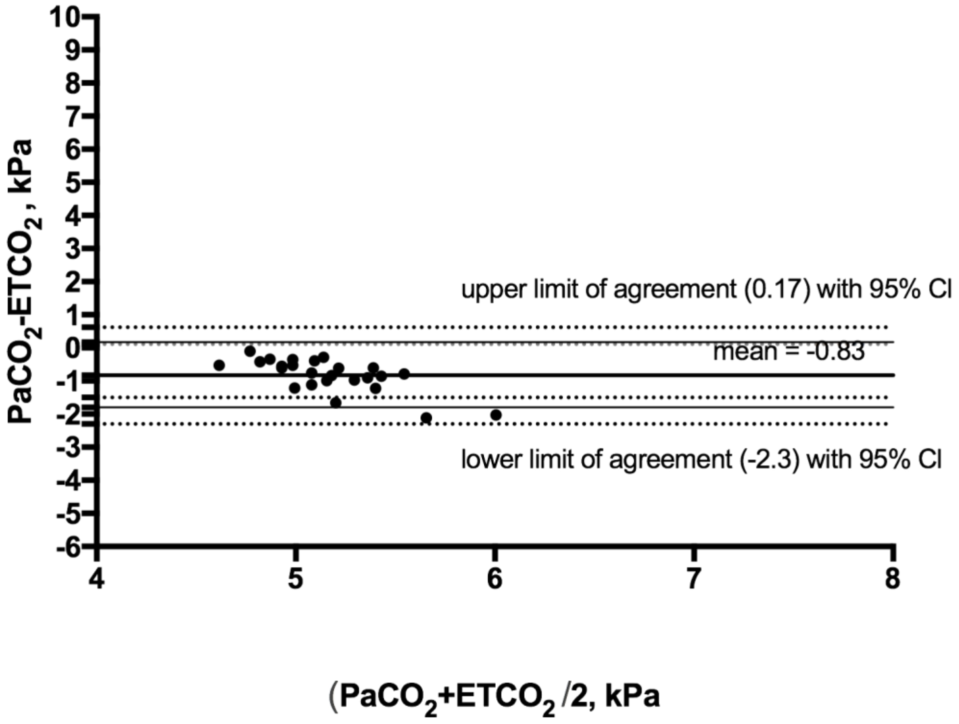

Table S1. Weight, interventions performed, number of ABG analyses, values of measured PaCO2 and pH values in the study subjects. The numbering of animals are based on timing, with the first experimental animal labelled number one and the last number nineteen.

Case no.	*Weight (kg)*	*Intervention*	*No. of ABG analyses*	*Lowest $PaCO_2$, kPa*	*Highest, $PaCO_2$, kPa*	*Mean $PaCO_2$, kPa*	*Highest pH*	*Lowest PH*	*Mean pH*
1	34	Metabolic acidosis	2	5.24	9.85	7.55	7.48	7.19	7.34
2	37	Metabolic alkalosis	10	4.92	6.81	6.21	7.66	7.36	7.53
3	29	CPR+defibrillation	2	4.82	6.72	5.77	7.47	7.33	7.40
4	30	Air embolism*	9	5.43	10.55	6.75	7.52	7.20	7.44
5	17,5	Metabolic acidosis	4	4.97	5.74	5.30	7.60	7.35	7.44
6	17,5	Left lung blocked**	5	5.28	7.02	6.13	7.49	7.40	7.44
7	46,5	Trendelenburg+NaCl***	5	5.31	6.27	5.74	7.48	7.42	7.47
8	31	Emergence	4	4.88	5.25	5.10	7.55	7.47	7.52
9	27	CO_2 insufflation****	5	5.29	6.81	6.14	7.41	7.50	7.47
10	25	CO_2 insufflation	4	5.16	6.53	5.96	7.48	7.41	7.43
11	19	CO_2 insufflation	3	5.65	6.16	5.93	7.46	7.43	7.45
12	23	CO_2 insufflation	4	5.64	6.42	5.95	7.50	7.46	7.48
13	21	CO_2 insufflation	4	5.6	6.67	6.21	7.44	7.39	7.41
14	26	CO_2 insufflation	3	5.21	6.13	5.75	7.82	7.49	7.50
15	15,5	CO_2 insufflation	4	5.81	13.75	8.40	7.50	7.18	7.44
16	25	CO_2 insufflation	3	5.86	6.08	5.94	7.50	7.49	7.49
17	47	CO_2 insufflation	3	5.41	5.95	5.68	7.53	7.51	7.52
18	25	CO_2 insufflation	3	5.6	6.2	5.88	7.53	7.50	7.52
19	51	CO_2 insufflation	6	5.86	7.41	6.54	7.52	7.44	7.49

ABG analysis= arterial blood gas analysis, CPR = cardiopulmonary resuscitation, PaCO2= arterial partial pressure of carbon dioxide

*Air embolism: injection of 60 ml of air into the central vein ** left lung blocked by placing a Swanz catether in the left bronchus ***400 ml of sodium chloride infused into lungs, ****CO2 insufflation (15 mmHg) to abdominal cavity for simulation of laparoscopic operation

Data about study subjects: weight, interventions, number of ABG analyses, lowest measured arterial partial pressure of carbon dioxide (PaCO2) value, highest measured PaCO2 value, mean of measured PaCO2 values, highest measured potential of hydrogen (pH) value, lowest measured pH value, mean of measured pH values.

Table S2. Hemodynamic variables, etCO2, etO2, PaO2 and PaO2/FiO2 ratios of each animal during the experiment.

	$PETCO_2$ (kPa), Median (IQR)	*$PETO_2$ (kPa), Median (IQR)*	*PaO_2 (kPa), Median*	*HR, Median (IQR)*	*MAP (mmHg), Median (IQR)*	*PaO_2/FiO_2 Ratio, Mean*	*FiO_2 %, Median (IQR)*	*PEEP, (cmH_2O) Median (IQR)*
**1**	5.37 (4.9-6.1)	33.9 (23.7-42.7)	10.05	119 (115-122)	116 (107-122)	248	36 (30-43)	2 (1-2)
**2**	5.31 (4.9-5.73)	32.0 (31.8-32.6)	24.51	116 (112-122)	93(77-107)	462	36 (36-37)	2 (2-3)
**3**	4.95 (3.18-5.05	29 (25.4-65.1)	24.92	118 (111-130)	85 (10-93)	553	32 (30-66)	2 (2-3)
**4**	5.04 (4.94-5.13)	37,63 (36.7-42.4)	8.95	115 (109-141)	93 (87-105)	277	42 (41-46)	5 (3-12)
**5**	5 (4.71-5.34)	33.4 (33.1-48.9)	28.61	117 (113-124)	51 (40-74)	554	38 (37-53)	3 (2-5)
**6**	5.68 (5.14-6.22)	34 (33.7-34.25)	24.40	139 (138-141)	125 (114-132)	650	39 (39-40)	2 (1-2)
**7**	4.98 (4.89-5.29)	35.5 (35.2-36.1)	27.16	96 (92-100)	89 (83-103)	475	40 (40-40)	8 (2-9)
**8**	5.02 (4.93-5.13)	35.2 (35-35.4)	27.61	106 (103-109	88 (81-109)	516	35 (35-35)	2 (2-2)
**9**	5.01 (4.96-5.25)	40.1 (36.6-40.8)	32.53	139 (136-140)	131 (129-134	430	44 (44-45)	2 (2-2)
**10**	5.16	34.4 (34.1-34.9)	26.23	126 (126-139)	98 (89-103)	543	39 (39-39)	5 (2-5)
**11**	5.38 (5.24-5.45	29.6 (29.4-29.9)	23.32	126 (117-130)	94 (87-109)	527	34 (34-34)	5 (5-5)
**12**	5.45 (5.2-6.02	25.23 (24.2-26.3)	24.49	105 (99-115)	89 (81-105)	500	30 (29-32)	5 (4-5)
**13**	5.1 (4.9-5.3)	24.9 (24.6-25.1)	16.25	95 (90-100)	85 (77-92)	437	29 (29-30)	5 (4-5)
**14**	5.12 (4.9-5.3)	30.1 (29.7-30.5)	23.10	120 (108-127)	91 (74-116)	491	35 (34-35)	5 (5-5)
**15**	5.39 (5.09-6.7)	25.2 (24.3-26.3)	18.28	92 (85-117)	89 (79-95)	482	31 (30-33)	5 (5-6)
**16**	5.22 (4.94-5.29)	24.8 (24.5-25.1)	18.55	127 (117-146)	84(76-99)	483	29 (28-29)	5 (2-5)
**17**	5.22 (4.96-5.33)	25.9 (25.0-26.2)	19.68	120 (120-136)	91 (78-101)	481	31 (30-31)	5 (5-6)
**18**	5.12 (5.04-5.27)	25.0 (24.9-25.3)	18.67	113 (106-130)	76 (74-85)	493	29 (29-30)	5 (5-5)

19	5.07	33.5 (25.1-48.8)	26.00	95 (93-104)	104 (93-113)	548	38 (29-52)	8 (6-8)

etCO2 = end-tidal carbon dioxide; etO2 = end-tidal oxygen; PaO2 = arterial partial pressure of oxygen; HR = heart rate; MAP = mean arterial pressure; FiO2 = fraction of inspired oxygen; PEEP= positive end-expiratory pressure.

Table S3. Estimation of PaO2 based on values of oxygen-dissociation curve.

SpO2(%)	PaO2(kPa)	SpO2(%)2	PaO2(kPa)
50	3,6	76	5,49
51	3,66	77	5,6
52	3,71	78	5,71
53	3,76	79	5,82
54	3,82	80	5,92
55	3,87	81	6,05
56	3,93	82	6,22
57	3,98	83	6,38
58	4,04	84	6,55
59	4,11	85	6,72
60	4,19	86	6,88
61	4,26	87	7,08
62	4,33	88	7,33
63	4,4	89	7,59
64	4,47	90	7,85
65	4,54	91	8,16
66	4,61	92	8,56
67	4,69	93	8,96
68	4,76	94	9,5
69	4,83	95	10,08
70	4,9	96	10,92
71	4,97	97	12,14
72	5,06	98	14
73	5,17	99	15,43
74	5,28	100	24
75	5,39		
